# Alpha Lipoic-Acid Potentiates Ex Vivo Expansion of Human Steady-State Peripheral Blood Hematopoietic Primitive Cells

**DOI:** 10.3390/biom12030431

**Published:** 2022-03-11

**Authors:** Christelle Debeissat, Maryse Avalon, Mathilde Huart, Pascale Duchez, Laura Rodriguez, Marija Vlaski-Lafarge, Zoran Ivanovic, Philippe Brunet de la Grange

**Affiliations:** 1 Etablissement Français du Sang Nouvelle Aquitaine, Place Amélie Raba Léon, CS22010, CEDEX, 33075 Bordeaux, France; cdebeissat@yahoo.fr (C.D.); maryse.avalon@efs.sante.fr (M.A.); mathilde.huart@u-bordeaux.fr (M.H.); pascale.duchez@efs.sante.fr (P.D.); laura.rodriguez@efs.sante.fr (L.R.); marija.vlaski@efs.sante.fr (M.V.-L.); zoran.ivanovic@efs.sante.fr (Z.I.); 2 Inserm Bordeaux UMR 1035, 33000 Bordeaux, France; 3 Department of Biological and Medical Sciences, Campus Carreire, University of Bordeaux, 33000 Bordeaux, France

**Keywords:** alpha lipoic acid, hematopoietic stem cells, hematopoietic progenitors, oxidative metabolism, Reactive Oxygen Species (ROS), Gluthatione (GSH)

## Abstract

Steady state peripheral blood (SSPB) contains hematopoietic stem and progenitor cells (HSPCs) presenting characteristics of real hematopoietic stem cells, and thus represents an interesting alternative cell supply for hematopoietic cell transplantation. Development of ex vivo expansion strategies could overcome the low HSPC numbers usually rescued from SSPB. We investigated the effect of alpha lipoic acid (ALA) on ex vivo culture of SSPB CD34 positive (CD34*^pos^*) cells on primitive cell expansion, cell cycle, and oxidative metabolism as estimated by determining the ROS and GSH content. ALA increased the ex vivo expansion of total CD34*^pos^* cells and of phenotypically defined CD34*^pos^* HSPCs subpopulations that retained in vivo repopulating capacity, concomitantly to a decreased expansion of differentiating cells. ALA did not modify cell cycle progression nor the proliferation of ex vivo expanded CD34*^pos^* cells, and coherently did not affect the ROS level. On the contrary, ALA decreased the proliferation and disturbed cell cycle progression of cells reaching a differentiated status, a phenomenon that seems to be associated with a drop in ROS level. Nonetheless, ALA affected the redox status of hematopoietic primitive cells, as it reproducibly increased GSH content. In conclusion, ALA represents an interesting molecule for the improvement of ex vivo expansion strategies and further clinical application in hematopoietic cell transplantation (HCT).

## 1. Introduction

Adult hematopoietic cell transplantation (HCT) currently represents a curative therapy for a large panel of hematological and non-hematological disorders. Nowadays, hematopoietic cells used for HCT are predominantly obtained from mobilized peripheral blood (mPB), as this source allows for a fast, complete, and long-term hematopoietic recovery, combined with a secure and well-tolerated conditioning process for the donor during cell explant. Consequently, bone marrow (BM) and cord blood (CB) represent alternative hematopoietic stem cell (HSC) sources used when mPB is not adapted. In clinic, the assessment of the quality of cell populations to be transplanted is based on their content of CD34*^pos^* cell population, which contain the majority of hematopoietic progenitors (HPs) and HSCs. While the vast majority of human HSCs resides in BM, a small proportion are normally released in steady state peripheral blood (SSPB). In the 1980s, a clinical study demonstrated the ability of SSPB to repopulate patients receiving myeloablative conditioning in an autologous context [1], but the low number of HSCs contained in SSPB implied the pooling of cells from several leukaphereses to achieve hematopoietic engraftment. The development of a mobilization regimen with hematopoietic growth factors at the end of the 1980s [2,3,4] stopped clinical investigations on SSPB in HCT, and progressively replaced conventional BM transplantation during the 1990s to become the reference method in auto-HCT. Nonetheless, despite the clinical efficiency of mPB in HCT and the global good tolerance of the mobilization regimen for the donor, PB mobilization can induce severe adverse effects in rare cases and nearly all donors experience inconveniences such as bone pain, headache, and nausea [5]. Therefore, as SSPB HSCs possess functional characteristics of real HSCs [6,7,8,9,10], they represent an easy and interesting source of HSCs for HCT, provided reliable ex vivo procedures to amplify HSC to numbers sufficient for an effective engraftment are developed. The ex vivo expansion of HSC without the loss of a marrow repopulating ability and long-term stem cell potential is a challenging question. Numerous studies have focused on CB ex vivo expansion strategies for transplantation [11], but due to biological discrepancies between the two sources of HSCs, one cannot assume that the conditions proven effective for CB will also be effective for SSPB. Thus, SSPB-specific studies are required.

Regulation of oxidative and energy metabolism has been demonstrated to be essential for HSCs homeostasis: HSC mainly relies on glycolysis for energy generation and presents a low mitochondrial activity, which is essential for stem cell maintenance [12,13]. The combination of a weakly oxygenated BM microenvironment [14] and faintly active mitochondria favors a low ROS content in HSCs, which supports their maintenance through cell cycle regulation [15,16,17,18]. In addition, Nrf-2, a major sensor/regulator of the cellular oxidative metabolism, is involved in the maintenance of HSC function [19], emphasizing the importance of energy metabolism in HSC biology. In line with this, several studies have demonstrated the capacity of exogenous antioxidant molecules to enhance the maintenance of HSC during ex vivo culture [20,21].

Alpha lipoic acid (ALA) is a naturally occurring molecule, synthesized in mammals directly and covalently bound to mitochondrial multi-enzymatic complexes implied in metabolism regulation. ALA is essential for the stabilization and biological activity regulation of mitochondrial enzymatic complexes involved in the tricarboxylic acid cycle, and thereby participates in cellular energy control. Exogenous ALA will be reduced in the cell and thereby acts as a potent antioxidant, directly scavenging free radicals or modulating different metabolic pathways. The in vitro supply of ALA in various models and cell types has been shown to protect cells against the toxicity of several chemical compounds that induce oxidative stress either through the Nrf2 pathway [22,23,24] and/or increased glutathione regeneration [25,26]. Moreover, ALA was recently shown to increase the hematopoietic reconstitution potential after ex vivo culture of the total CB cells [27]. ALA thus represents a good candidate to support the maintenance of the stem cell potential in the ex vivo culture of SSPB.

In this study, we assessed the effects of ALA on the ex vivo expansion of SSPB primitive hematopoietic cells. We observed that ALA treatment allowed for an increase in the total numbers of CD34*^pos^* cells accompanied by the maintenance or even the tendency to increase phenotypically and functionally defined HSCs during the ex vivo culture, but it also increased the primitive hematopoietic progenitor content. We explored the oxidative metabolism to further understand the mechanisms implied in this phenomenon and showed that, despite no mitochondrial or cytoplasmic ROS level modification in the primitive CD34*^pos^* population, the presence of ALA in ex vivo culture induced a trend towards intracellular GSH increase, consequently affecting the physiological oxidative metabolism of primitive hematopoietic cells.

## 2. Materials and Methods

### 2.1. CD34^pos^ Isolation from Steady State Peripheral Blood

SSPB mononuclear cells (MNCs) were retrieved from leukoreduction filters (LRF; LEUCOFLEX, Macopharma, Tourcoing, France) with an extraction buffer consisting in 0.2% glucose and 0.9% NaCl solution supplemented with 8.3% ACD-A (*Ref. ABS0734EU*, Macopharma) and 25% human plasma, loaded on Ficoll and centrifuged (HERAEUS Multifuge X3R, Thermo Scientific, Illkirch, France) 23 min at 930× *g*. MNC were recovered and washed first with sucrose for 15 min at 1200× *g* and then twice with selection buffer-PBS −1.4% albumin-2 mM EDTA. The cells were incubated for 5 min in Pulmozyme and labeled with “EasySep^TM^ Human CD34 positive kit II” (Stem Cell Technologies, Grenoble, France): the cells were incubated 10 min at +4 °C with “EasySep^TM^ human CD34 positive selection cocktail” (30 µL for each LRF), and for an additional 10 min at +4 °C with “EasySep^TM^ dextran rapid spheres^TM^ 50100” (20 µL for each LRF). The cells were then washed with a selection buffer and loaded on an LS column inserted in the MidiMACS separator (Miltenyi Biotec, Paris, France). Magnetically retained CD34*^pos^* cells were then flushed with Stem Span^TM^ SFEM II (Stem Cell Technologies), counted with trypan blue exclusion dye, and processed for experiments.

### 2.2. Cell Culture

CD34*^pos^* cells were cultured at 10^5^ cells/mL in Stem Span^TM^ SFEM II supplemented with 100 ng/mL rhSCF (Peprotech, Neuilly-sur-Seine, France), 100 ng/mL rh Flt-3L (Peprotech), 20 ng/mL rhTPO (Peprotech), and 10 ng/mL rhG-CSF (Neupogen; AMGEN, Boulogne Billancourt, France). The cells were diluted in a fresh medium at days 3 and 6 to maintain the cell concentration under 1.5 × 10^6^ cells/mL, and were analyzed at the time points specified in the Results section.

### 2.3. Immunophenotyping

The phenotype of primitive hematopoietic cells was evaluated at different time points during the ex vivo expansion based on the cell membrane expression of specific antigens. The cells were harvested from the culture, centrifuged (HERAEUS Multifuge X3R, Thermo Scientific, Illkirch, France) at 300× *g* for 5 min, and labeled 15 min at room temperature (RT) with BV480 anti-human CD45RA (BD Biosciences, Le Pont de Claix, France), APC-Alexa Fluor 750 anti-human CD34 (Beckman Coulter, Villepinte, France), PE anti-human CD133 (BD Biosciences), and 7AAD (Exbio-Clinisciences, Nanterre, France). The cells were washed with PBS, centrifuged for 5 min at 300× *g* and analyzed by flow cytometry with a FACS Canto II (BD Biosciences). The FACS data were processed with FlowJo^TM^ software version 10.8.0 (BD Biosciences).

### 2.4. Apoptosis

Apoptosis was evaluated during the expansion culture based on extra-membrane detection of phosphatidylserine with AnnexinV-FITC kit (IM3546, Beckman Coulter, Villepinte, France), according to the manufacturer’s instructions. Three hundred thousand cells were washed with PBS, resuspended in 100 µL of the binding buffer provided in the kit, and incubated for 15 min on ice with 1 µL AnnexinV-FITC and 10 µL PI. After incubation, the cells were maintained on ice, diluted with 300 µL binding buffer, and immediately analyzed by flow cytometry with a FACS Canto II. FACS data were processed with FlowJo^TM^ software version 10.8.0 (BD Biosciences).

### 2.5. Mice

NSG mice were obtained from the Jackson laboratory and bred in-house. Female NSG mice from 8 to 12 weeks old were used for all of the experiments.

### 2.6. SCID-Repopulating Cells (SRC) Limiting Dilution Assay

The mice were conditioned by two intra-peritoneal injections of 20 mg/kg busulfan (Busilvex^®^; Pierre Fabre, Boulogne, France), at 24 h intervals. One to seven days after the second injection of busulfan, the mice were injected by the intravenous route with the cell progeny of 10,000, 5000, or 2500 initially—time 0-seeded CD34*^pos^*. Twelve weeks after transplantation, the mice were sacrificed and the femoral BM was harvested from the femurs. The total BM cells were labeled after 15 min at RT with BV711 BV421 anti-murine CD45, FITC anti-human CD45, PE anti-human CD19, and APC anti-human CD33. Human vs. mouse chimerism was analyzed by flow cytometry with FACS Canto II. 

The SRC frequency was determined using extreme limiting dilution analysis (ELDA) webtool accessible at http://bioinf.wehi.edu.au/software/elda/ (accessed on 26 January 2022) [28]. 

### 2.7. Proliferation Analysis

Cell proliferation was evaluated by Carboxy Fluorescein Succinimidyl Ester (CFSE) labeling. For this purpose, CD34*^pos^* cells were labeled at time 0 immediately after their sorting from LRF. The CD34*^pos^* cells were washed twice with PBS, resuspended at 1.10 × 10^6^ cells/mL in PBS, and incubated with 1 µM CFSE (ThermoFisher Scientific, Illkirch, France) for 10 min at 37 °C. The cells were then washed twice with PBS (300× *g*, 5 min) and a third time with Stem Span^TM^ SFEM II. The CFSE labeled cells were incubated overnight at +4 °C before use, to allow for the excess unconjugated reagent to leave the cells. The level of fluorescence was evaluated by flow cytometry with FACS Canto II at time 0 and during the first 5 days of culture. FACS data were processed with FlowJo^TM^ software version 10.8.0.

### 2.8. Cell Cycle Analysis

The progression of cultured cells through the cell cycle phases—G0, G1, S, and G2/M—was evaluated thanks to Ki67 and 7AAD labeling. For this purpose, 1.10 × 10^6^ cells were centrifuged (HERAEUS Multifuge X3R, Thermo Scientific, Illkirch, France) at 300× *g* for 5 min, resuspended in PBS, and incubated with BV421 anti-human CD34 for 15 min at RT. The cells were then washed twice with PBS, resuspended with 100 µL PBS, and incubated for 30 min at RT with 900 µL of fixation buffer—formaldehyde 0.4%, saponin 0.03%, and HEPES 1 mM. Cells were next washed at 300× *g* during 5 min and resuspended with washing buffer—PBS, FBS 2%, saponin 0.03%—and incubated with Alexa Fluor 647 anti-human Ki67 and 7AAD for 30 min at RT. Finally, the cells were washed with PBS at 300× *g* for 5 min and fluorescence collected with a FACS Canto II. FACS data were processed with FlowJo^TM^ software version 10.8.0.

### 2.9. Mitochondrial and Cytoplasmic ROS Level Analysis

The ROS levels in the mitochondria and cytoplasm were evaluated by labeling with the MitoSOX red mitochondrial superoxide indicator (ThermoFisher Scientific, Illkirch, France) and the CellROX^TM^ deep red reagent (ThermoFisher Scientific), respectively.

For the mitochondrial ROS evaluation, 1.10 × 10^5^ cells were washed with HBSS, resuspended at 1.10^6^ cells/mL in HBSS-5 µM MitoSOX, and incubated 10 min at 37 °C. Cells were washed three times with HBSS, resuspended in PBS, incubated with BV421 anti-human CD34 and APC anti-human CD133 for 15 min at RT. The cells were then washed with PBS and the fluorescence data immediately collected with FACS Canto II. 

For the cytoplasmic ROS evaluation, 1.10^5^ cells were incubated 30 min at 37 °C in 500 µL Stem Span^TM^ SFEM II-5 µM CellROX. After incubation, the cells were washed three times with PBS, resuspended in PBS, and incubated with BV421 anti-human CD34 and PE anti-human CD133 for 15 min at RT. After incubation, the cells were washed with PBS and the fluorescence data were immediately collected with FACS Canto II.

FACS data were processed with FlowJo^TM^ software version 10.8.0.

### 2.10. GSH and GSSG Determination

The reduced (GSH) and oxidized (GSSG) forms of glutathione were determined by luminescence assay using a GSH/GSSG-Glo^TM^ Assay (Promega, Charbonnières-les-Bains, France), according to the manufacturer’s instructions. The cells were harvested at different time-points of culture, and 5000 CD45RA*^neg^*CD34*^pos^*CD133*^pos^*, CD45RA*^neg^* CD34*^pos^*CD133*^neg^*, or CD45RA*^pos^* CD34*^pos^*CD133*^pos^* cells were FACS-sorted with a FACS Melody (BD Biosciences), centrifuged (HERAEUS Multifuge X3R, Thermo Scientific, Illkirch, France), and resuspended in 25 µL HBSS before loading on a 96-well luminometer-compatible plate. Twenty-five microliters of total glutathione lysis reagent (for total glutathione measurement) or oxidized glutathione lysis reagent (for oxidized glutathione measurement) were added to each well, and the plate was kept under shaking for 5 min at RT. After incubation, 50 µL of the luciferin generation reagent were added and incubated once more for 30 min at RT. Finally, 100 µL of the luciferin detection reagent were added and incubated for 15 min, and the luminescence was recorded with a GloMax luminometer (Promega, Charbonnières-les-Bains, France) with a light signal integration time of 1 s. Each biological sample was run in three technical replicate wells. For analysis, the GSH level was the result of total GSH—oxidized GSSG.

### 2.11. Statistical Analysis

Statistical analysis was performed under R environment with RStudio software version 2021.09.0+351. In vitro data are presented as arithmetic mean ± SEM, and Wilcoxon or Friedman tests were performed to evaluate the statistical significance. In vivo data are presented as median for each group of mice, and the Kruskal–Wallis test followed by Dunn post-hoc test were performed to evaluate statistical significance. 

## 3. Results

### 3.1. ALA Allows Expansion of Phenotypically-Defined Primitive Cells

**Figure 1:**
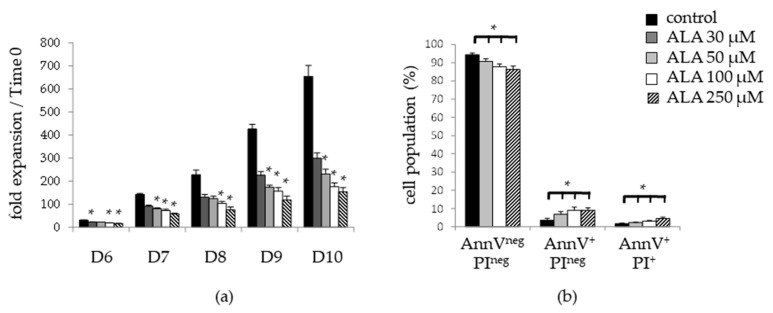
ALA decreases the total cell expansion of SSPB CD34*^pos^* cells over 10 days of ex vivo culture. Ex vivo culture of SSPB CD34*^pos^* were performed in the presence of 0, 30, 50, 100, or 250 µM ALA over 10 days. (**a**) Total cell growth of SSPB CD34*^pos^* was evaluated from D6 to D10 by trypan blue exclusion, and expressed as fold expansion relative to time 0 (*n* = 4–6). (**b**) Apoptosis analyzed by flow cytometry after AnnV/PI labeling at D5 (*n* = 4). * *p* < 0.05.

We preliminary tested the effect of 30 to 250 µM ALA concentrations on the ex vivo expansion of SSPB CD34*^pos^* cells over 10 days. We observed a dose-dependent decrease in the total cell expansion in the presence of ALA (Figure 1a). This event was not due to apoptosis as we observed only a slight induction of apoptosis even at the highest ALA dose of 250 µM (9.1 ± 1.4% AnnexinV^+^/PI*^neg^* and 4.5 ± 0.8% AnnexinV^+^/PI^+^) compared to the control condition (4.9 ± 1.5% AnnexinV^+^/PI^neg^ and 2.2 ± 0.3% AnnexinV^+^/PI^+^) (Figure 1b).

**Figure 2:**
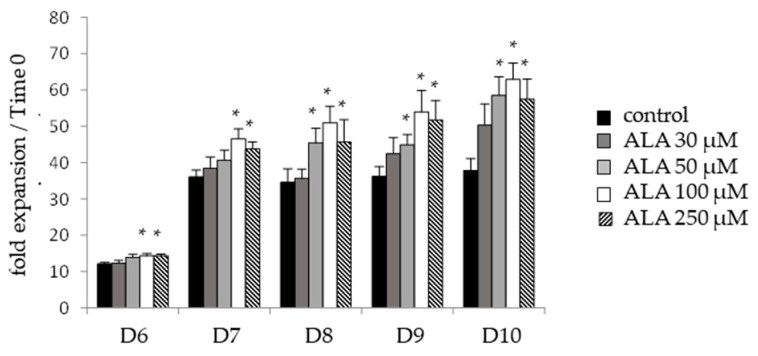
ALA specifically increases the expansion of the CD34*^pos^* compartment during the ex vivo culture of SSPB CD34*^pos^* cells. SSPB CD34*^pos^* cells were cultured in the presence of 0, 30, 50, 100, or 250 µM ALA. The CD34*^pos^* cell content is evaluated from D6 to D10 by flow cytometry after CD34 labeling, and is expressed as fold expansion relative to time 0 (*n* = 4–6). * *p* < 0.05.

Interestingly, focusing on the criteria used to qualify hematopoietic graft in the context of cell therapy, i.e., CD34*^pos^* cells, we demonstrated a significant increased expansion of this primitive CD34*^pos^* population, reaching a peak after 10 days for 50 µM, 100 µM, and 250 µM ALA with 55%, 66%, and 52%, respectively, increased expansion compared to the control condition (Figure 2).

**Figure 3:**
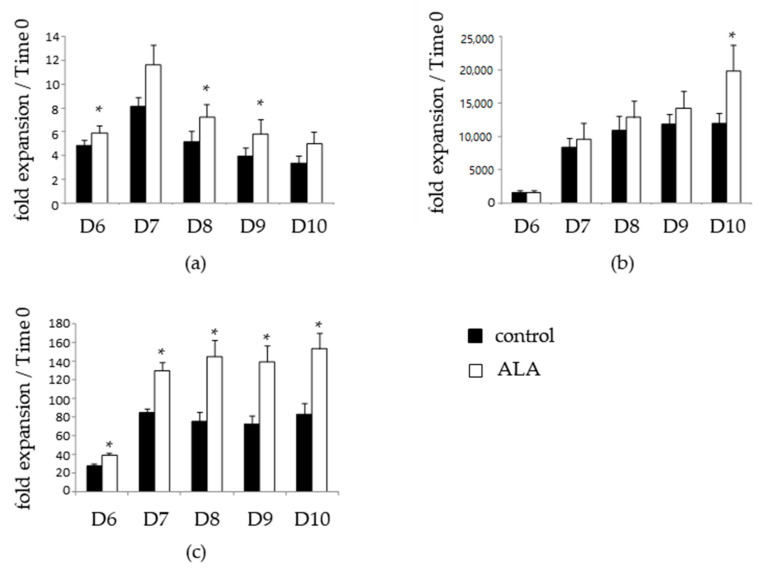
ALA increases the expansion of phenotypically defined primitive hematopoietic populations during ex vivo culture. SSPB CD34*^pos^* cells cultured for 10 days in the presence of 0 or 100 µM ALA. Analysis of (**a**) HSC/MPP, (**b**) EMP, and (**c**) LMP populations assessed at D6, D7, D8, D9, and D10 through a combination of CD45RA, CD34, and CD133 labeling and flow cytometry analysis, and expressed as fold expansion relative to time 0 (*n* = 6). * *p* < 0.05.

The following analyses were conducted at 100 µM ALA, which provided the highest CD34*^pos^* cell expansion. We next performed a deeper phenotypic analysis combining CD45RA, CD133, and CD34, which allowed for detecting the HSC/multipotent progenitors (HSC/MPP; CD34*^pos^*, CD45RA*^neg^*, and CD133*^pos^*), lymphoid-primed multipotent progenitors (LMP; CD34*^pos^*, CD45RA*^pos^*, and CD133*^pos^*), and erythro-myeloid progenitors (EMP; CD34*^pos^*, CD45RA*^neg^*, and CD133^*low*^) [29]. The expansion of HSC/MMPs peaked at D7 of the ex vivo culture (Figure 2a), while the expansion of EMP (Figure 2b) and LMP (Figure 2c) gradually increased until D10. ALA induced a significant increase in the expansion of these three hematopoietic lineages compared to the control condition (Figure 3a–c), confirming the ALA capacity to ex vivo preserve hematopoietic cell primitiveness.

### 3.2. ALA Increases SRC Frequency after 10 Days of Culture

**Figure 4:**
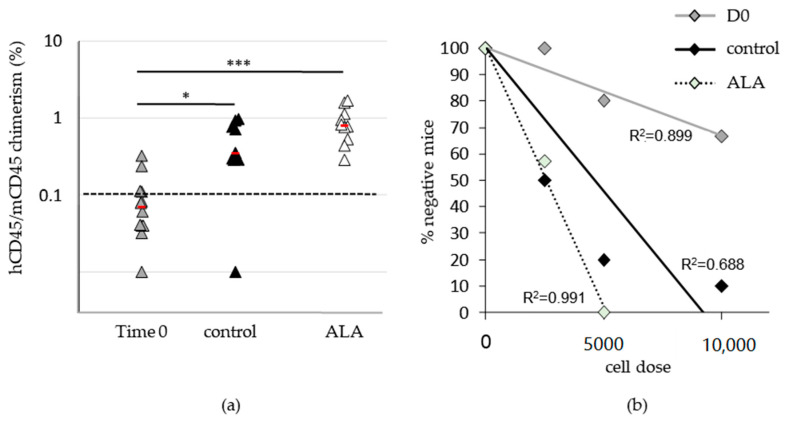
ALA favors the expansion of cells retaining a hematopoietic reconstitution potential. Freshly isolated time 0 or 10 day-expanded 10,000 SSPB CD34*^pos^* cells in the absence or presence of 100 µM ALA injected in busulfan-conditioned mice and BM cells analyzed 12 weeks after injection by flow cytometry after mCD45/hCD45 co-labeling. (**a**) Mouse vs. human chimerism; * *p* < 0.05, *** *p* < 0.0005. (**b**) Percentages of negative mice plotted as a function of the cell dose injected.

We next analyzed the effects of ALA on cells retaining the ability to reconstitute hematopoiesis in vivo, i.e., on SRC. The frequency of SRC was evaluated through injection of the total progeny of 10,000, 5000, and 2500 time 0-SSPB CD34*^pos^* cells after 10 days of culture. As shown in Figure 4, we first of all confirmed previous data [8] showing that ex vivo culture of SSPB CD34*^pos^* cells increased the SSPB repopulation potential, as we observed a significantly higher hCD45/mCD45 ratio in the mice infused with D10 control-expanded cells compared to the freshly isolated cells (Figure 4a). In this context, ALA-treated cultures exhibited a trend toward an increased repopulation potential compared to the control condition-expanded cells. Notably, the progeny of 10,000 time 0 cells provided a hCD45/mCD45 median chimerism of 0.808% vs. 0.352% for the ALA and control conditions, respectively (Figure 4a). We then performed a limiting dilution assay through the injection of different cell doses to mice (Figure 4b), and estimated SRC frequencies thanks to the ELDA webtool in each condition (Table 1). In this context, we obtained SRC frequencies of 1/19,390 for time 0 SSPB CD34*^pos^* cells, 1/3796 for D10 control-expanded cells, and 1/2485 for D10 ALA-expanded cells. Thus, the ex vivo expansion of SSPB CD34*^pos^* cells in the presence of ALA tends to improve the expansion of cells retaining a hematopoietic reconstitution potential compared to the control condition.

### 3.3. ALA Modifies Proliferation and Cell Cycle Distribution of CD34^neg^ Population but Not of CD34^pos^

**Figure 5:**
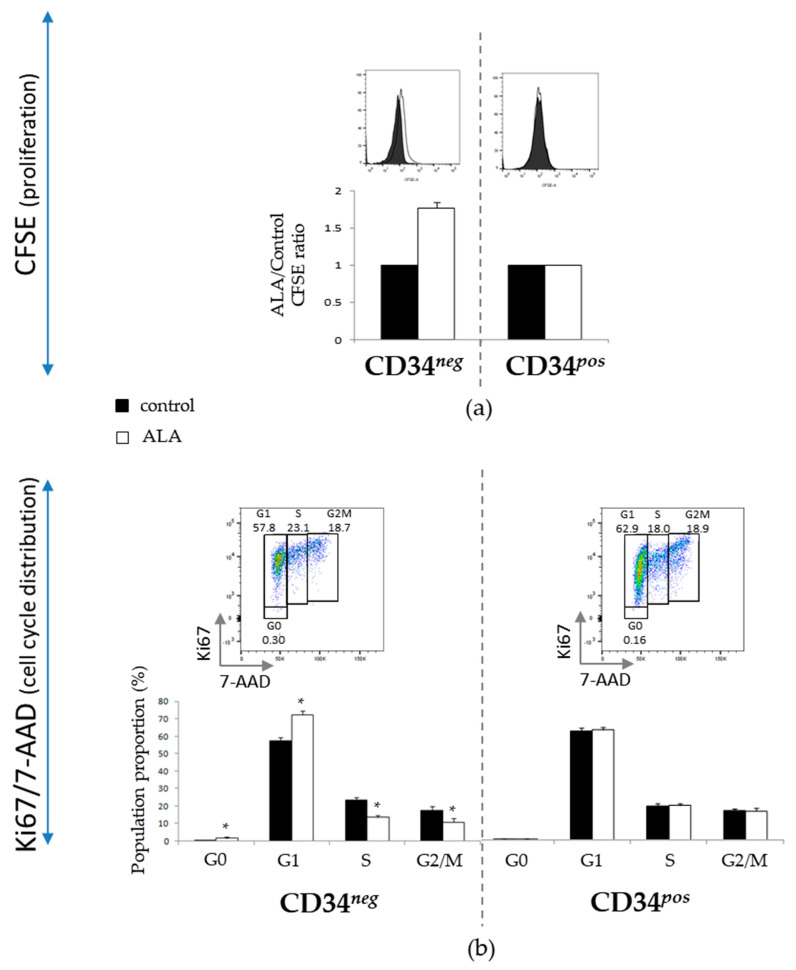
ALA does not modify the proliferation or cell cycle repartition of ex vivo expanded SSPB CD34*^pos^* cells (*n* = 5). (**a**) Analysis of proliferation in CD34*^pos^* and CD34*^neg^* populations. SSPB CD34*^pos^* cells labeled with CFSE at time 0 and cultured for 5 days in the absence or the presence of 100 µM ALA. The proliferation of expanded CD34*^neg^* and CD34*^pos^* cells evaluated by flow cytometry after CD34 labeling (*n* = 3); upper panel: representative experiment for CD34*^neg^* and CD34*^pos^* populations. (**b**) Cell cycle performed by flow cytometry after CD34/Ki67/7AAD co-labeling (*n* = 5); upper panels: representative experiments. * *p* < 0.05.

To gain insight into the cellular mechanisms implied in primitive hematopoietic cell preservation by ALA, we analyzed the proliferation kinetic (number of cell divisions) and cell cycle distribution by CFSE and Ki67/7AAD labeling, respectively. We observed that after 5 days of culture, ALA induced a decreased proliferation kinetic in the CD34*^neg^* population (Figure 5a) associated with an increased proportion of cells in G0 and G1 phases at the expense of proliferative S and G2/M phases (Figure 5b). In contrast, ALA did not modify CD34*^pos^* cells’ proliferation kinetic (Figure 5a) or cell cycle distribution (Figure 5b). The total cell decrease, previously reported in Figure 1, in the presence of ALA therefore reflects the decreased expansion of the more differentiated CD34*^neg^* population, without modification of the CD34*^pos^* population proliferation’s kinetic (Figure 5a), in spite of the increased number of CD34*^pos^* cells.

### 3.4. ALA Doest Not Modify Intracellularros Status in CD34^pos^ Cells but Increases Their GSH Content

**Figure 6:**
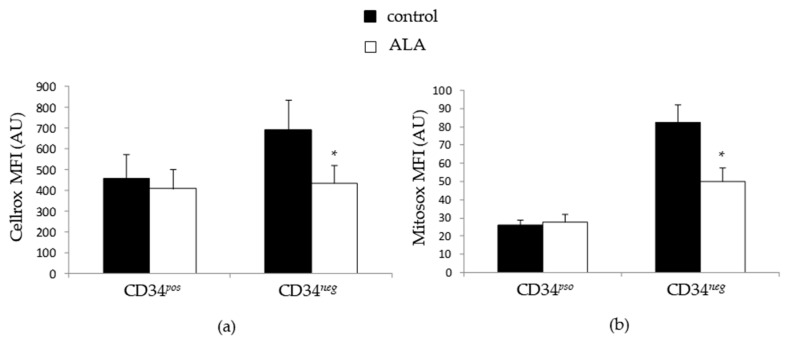
ALA does not modify the mitochondrial and cytoplasmic ROS level in the CD34*^pos^*-expanded population. SSPB CD34*^pos^* were cultured for 5 days in the absence or the presence of 100 µM ALA, and analyzed for (**a**) cytoplasmic ROS content by flow cytometry following CellROX labeling (*n* = 5) or (**b**) mitochondrial ROS content by flow cytometry following MitoSOX labeling (*n* = 4). * *p* < 0.05.

Due to the antioxidant nature of ALA and the importance of oxidative metabolism in HSC biology, we analyzed the mitochondrial and cytoplasmic ROS in the course of incubation. We observed that both the mitochondrial and cytoplasmic ROS levels were higher in the CD34*^neg^* population compared to the CD34*^pos^* one. Interestingly, the ROS analysis after 5 days of culture did not reveal any difference for cytoplasmic (Figure 6a) or mitochondrial ROS levels (Figure 6b) between the control and ALA-treated conditions in the CD34*^pos^* population, while ALA induced a marked reduction of both cytoplasmic and mitochondrial ROS levels in the CD34*^neg^* population. Consequently, the significantly higher CD34*^pos^* cell expansion that we observed in the presence of ALA seemed to be unrelated to the direct intracellular ROS-scavenging effect of ALA.

**Figure 7:**
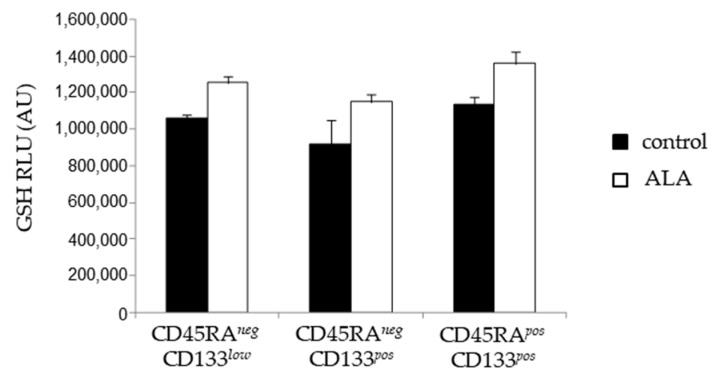
ALA increases the GSH content in HSC/MPP, LMP, and EMP populations. SSPB CD34*^pos^* cultured for 5 days in the absence or the presence of 100 µM ALA, and FACS-sorted for each CD34*^pos^* cell phenotype and analyzed for the GSH content.

In numerous studies [24,25,30], ALA was shown to modulate the oxidative metabolism through an increase in the intracellular GSH level. To gain insight into how ALA could be involved in the redox metabolism during the ex vivo expansion of CD34*^pos^* hematopoietic cells, we analyzed the GSH content at D5 in the three primitive populations. We observed in HSC/MMP, LMP, and EMP, previously defined as a non-statistically significant but reproducible increased level of GSH (Figure 7a) in the presence of ALA.

## 4. Discussion

SSPB represents an interesting source of HSCs for HCT, as it is easily accessible and safe for donors. However, one of the limitations of its use is the low CD34*^pos^* cell number collected in a single apheresis. This implies developing ex vivo protocols allowing for both the expansion of hematopoietic progenitors that support rapid (but transient) hematopoietic recovery in recipients, and the maintenance/increase of HSCs ensuring a long-term reconstitution potential. Therefore, successful grafting is directly linked to the quality of the transplanted cell population, which is evaluated in clinic through the analysis of its CD34*^pos^* content.

**Figure 8:**
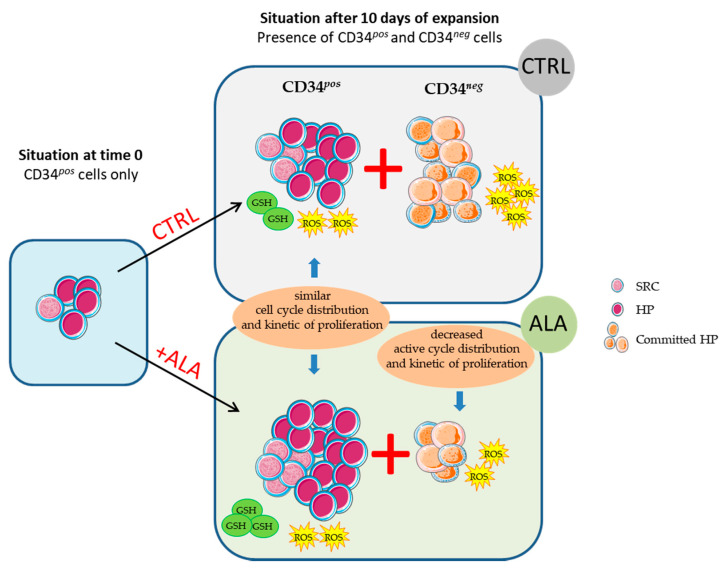
Overall representation of the main results of the study.

In this study, we report the beneficial effect of ALA to significantly increase the expansion of SSPB CD34*^pos^* cells in ex vivo culture. The main results of the study are summarized in Figure 8. 

ALA effects were notably observed in the most primitive CD34*^pos^* CD45RA*^neg^* CD133^*low*^HSC/MPP population, as well as in CD34*^pos^* CD45RA*^neg^* CD133*^pos^* EMP and CD34*^pos^* CD45RA*^pos^* CD133*^pos^* LMP committed progenitors. The SRC assay analysis was in line with these results and showed that ALA not only increased CD34*^pos^* cells, but also concomitantly preserved, and even extended, the engraftment potential after 10 days of ex vivo culture. Interestingly, our results showed that in SSPB, the effect of ALA is higher on MPP than on SRC, highlighting the potential interest in the use of ALA during cell preparation so as to achieve rapid recovery in patients thanks to increased HP numbers, without losing the potential of long-term recovery ensured by SRC. These data supplement previous work on human pluripotent stem cells (hPSCs) and cord blood cells, which showed that ALA was able to favor the regeneration of hematopoietic stem/progenitor cells derived from hPSCs, and better maintain the in vivo reconstitution potential of cord blood cells after ex vivo culture [27]. In view of deciphering the mechanism involved in such an effect, we then observed that ALA decreased the proliferation of the differentiated CD34*^neg^* population that accumulated in the G0/G1 phases at the expense of the S and G2M phase. The antiproliferative effect of exogenous ALA in ex vivo culture has been previously highlighted in numerous studies [31,32,33], and is often associated with a decrease in ROS content [27,34,35]. This is consistent with the need for a physiological increase in ROS for cell cycle progression from the G1 to S phase, already described [36]. Here, ALA did not modify the cell cycle distribution or cell division number in the CD34*^pos^* population, sustaining that ALA did not stimulate the proliferation kinetic of primitive cells but rather affected the nature of cell division and consequently oriented cell fate decision towards self-renewal through pathways that need to be dissected.

Considering the importance of redox status in proliferation/quiescence and differentiation/self-renewal balances of hematopoietic cells [37,38], we next investigated the cytoplasmic and mitochondrial ROS levels in the course of the culture. Our data showed that ALA differentially affected hematopoietic cells according to their differentiation state. We observed indeed an antioxidant effect of ALA on the CD34*^neg^* population, with a decrease of both mitochondrial and cytoplasmic ROS levels, while ALA did not affect ROS status in the more primitive CD34*^pos^* population. These observations were consistent with the aforementioned effect of ALA on cell proliferation. Indeed, the diminution of ROS observed in the presence of ALA in the CD34*^neg^* population was in line with its decreased proliferation through cell cycle reduction, while the unmodified proliferation of primitive hematopoietic CD34*^pos^* cells matched with the equal ROS content observed between the control and ALA condition. In addition, the lower ROS content in CD34*^pos^* compared to the CD34*^neg^* population was coherent with the low metabolic activity observed in the most primitive hematopoietic cells, while differentiation induced metabolic changes, including an increase of oxidative phosphorylation accompanied by a rise of ROS level [39]. Taken together, we observed that ALA slowed down the expansion of SSPB differentiating hematopoietic cells through ROS attenuation and favored primitive hematopoietic expansion in ex vivo culture in a ROS-independent mechanism. The differential effects of ALA on ROS content depending on hematopoietic differentiation state is of particular interest and needs further investigations.

While ALA can display its antioxidant effect through direct ROS scavenging, the recycling of the intracellular antioxidant, in particular GSH, is also reported [24,25,30]. To address in better detail the question of the mode of action of ALA in our expansion model, we determined the intracellular GSH content in the course of the culture and showed that ALA reproducibly increased GSH level in all CD34*^pos^* subpopulations, namely HSC/MPP, LMP, and EMP. The GSH-GPx system was shown to be essential for HSC preservation through a reduction of peroxydation [40], a fact that could explain the increased primitive hematopoietic cell expansion observed in our study. Several pathways were highlighted to induce GSH rise, including Nrf2 activation [24,25] and the increased intracellular availability of the reduced form of cysteine [30]. The mechanisms implied in GSH rise observed in our study remain to be elucidated.

Overall, further experiments are needed to improve the understanding of the ability of ALA to favor HSC/MPP self-renewal and therefore to expand primitive SSPB hematopoietic cells ex vivo. However, ALA represents an indisputable interesting molecule to increase the CD34*^pos^* cell content in hematopoietic grafts. Therefore, ALA could be used for therapeutic purposes in the ex vivo culture of SSPB primitive cells, potentially in combination with other described molecules, in the context of HCT.

## Figures and Tables

**Table 1:** ALA increases the SRC frequencies of ex vivo expanded SSPB CD34*^pos^*. Freshly isolated or the progeny of 10 days-expanded 10,000, 5000, and 2500 SSPB CD34*^pos^* cells, in the absence or the presensence of 100 µM ALA, were injected in busulfan-conditioned mice. BM hCD45 chimerism analyzed 12 weeks after injection by flow cytometry, after mCD45/hCD45 co-labeling and SRC frequencies calculated with ELDA webtool.

SRC Frequency (1/…)				Pairwise Test (ELDA Webtool)				
Group	Lower	Estimate	Upper	Group 1	Group 2	Chisq	DF	Pr (>Chisq)
Time 0	64,247	26,736	11,126	Control	Time 0	16.6	1	4.51 × 10^−5^
Control	6643	3796	2169	ALA	Time 0	23.2	1	1.44 × 10^−6^
ALA	4596	2485	1343	ALA	Control	0.972	1	0.324

## Data Availability

All data are conserved at the internal server of EFS-NVAQ Bordeaux and are available upon request.
